# Supplementary material for: Assembly of the threespine stickleback Y chromosome reveals convergent signatures of sex chromosome evolution
Source: Genome Biol. 2020 Jul 19;21:177. doi: 10.1186/s13059-020-02097-x (PMC7368989; doi:10.1186/s13059-020-02097-x)
Supplement: Supplementary file 1 — Additional file 1. Supplementary tables. [file 13059_2020_2097_MOESM1_ESM.pdf]

Supplemental Table 1. Sequencing statistics before and after assembly with Canu.

	PacBio preassembled reads	Canu assembly
**Total sequence length**	34.84 Gb	622.30 Mb
**Total contigs**	3,255,924	3593
**Contig N50 length**	21,068 bp	541,786 bp
**Max. contig length**	87,617 bp	12,930,346 bp
**Min. contig length**	1000 bp	1028 bp
**Mean contig lenth**	10,701 bp	173,198 bp
**Median contig length**	5969 bp	56,654 bp

Supplemental Table 2. Nucleotide substitutions per site between three different populations and the reference genome assembly (Bearpaw Lake) and the reference Y chromosome assembly (Paxton Lake).

**Population**	**Autosomes (Bearpaw)**	**X (Bearpaw)**	**Y (Paxton)**
**Puget Sound**	0.0028	0.0022	0.00014
**Lake Washington**	0.0025	0.0022	0.00015
**Paxton Lake benthic**	0.0038	0.0031	0.000025

Supplemental Table 3. Log_2 fold change between testis tissue and three other tissues for genes that are duplicated on the Y chromosome and have an X-linked gametolog.

Comparison	Y duplicated	X homolog	P value
**Testis versus Liver**	-1.300	-0.186	0.025
**Testis versus Brain**	-3.379	-0.114	0.006
**Testis versus Larvae**	-5.395	-4.872	0.998

Supplemental Table 4. The effect of sequence identity threshold on the total number of X chromosome contigs identified.

Sequence identity threshold	Number of X chr. contigs	Total contig length	Difference from reference X chr.
94%	133	23,137,131 bp	2,518,665 bp
95%	124	21,909,126 bp	1,290,660 bp
96%	114	21,255,474 bp	637,008 bp
97%	90	18,109,484 bp	-2,508,982 bp
98%	73	15,892,396 bp	-4,726,070 bp

Supplemental Table 5. The effect of the 3D-DNA parameter --editor-repeat-coverage on the number of concordant BACs that align to the assembly.

Editor repeat coverage	Number of concordant BACs
8	62
9	48
10	89
11	92
12	92
13	92
14	89
15	89
16	89
17	88
18	88
