# Supplementary material for: Assembly of the threespine stickleback Y chromosome reveals convergent signatures of sex chromosome evolution
Source: Genome Biol. 2020 Jul 19;21:177. doi: 10.1186/s13059-020-02097-x (PMC7368989; doi:10.1186/s13059-020-02097-x)

Figure S1. Canu assembled contigs were aligned to the reference genome. Contigs that aligned to the autosomes form a clear unimodal distribution, whereas contigs aligned to the X chromosome do not. The increased number of contigs aligned to the X chromosome with lower sequence identity were separated as putative Y chromosome contigs.

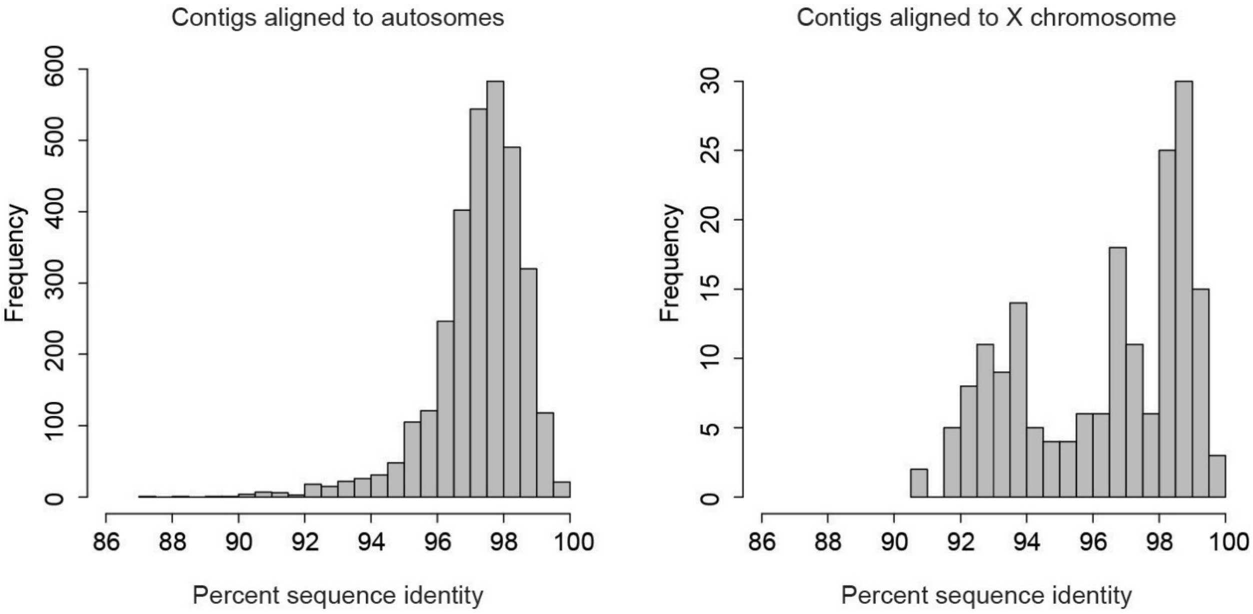

Figure S2. The PacBio assembly pipeline accurately reconstructed the X chromosome. The PacBio assembled X chromosome was split into three main scaffolds, with the two smallest scaffolds corresponding to the pseudoautosomal region and the third larger scaffold mostly aligning to the remainder of the reference X chromosome. Alignments between the PacBio assembled X chromosome and the reference X chromosome are largely colinear. Positions are shown in megabases.

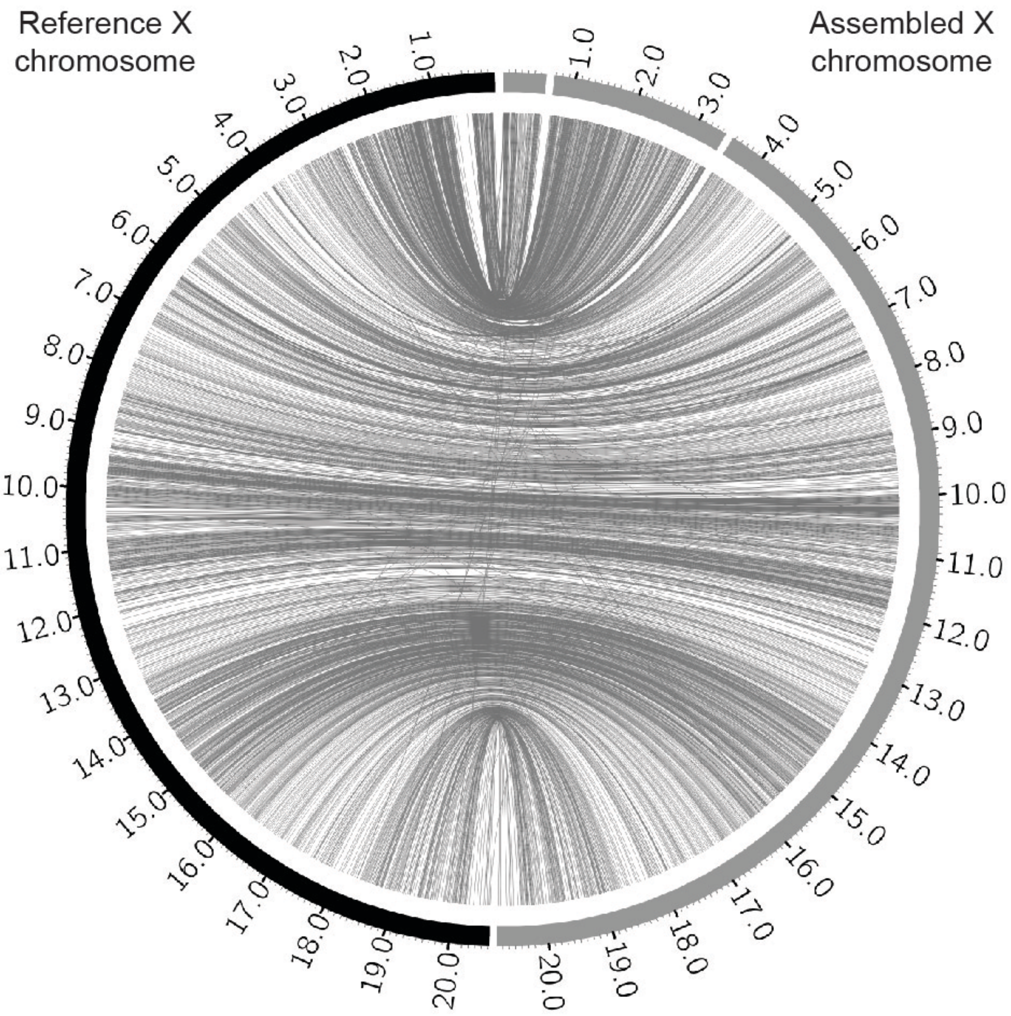

Figure S3. Hi-C chromosome conformation capture sequencing generated a single Y chromosome scaffold. The contact matrix shows a mis-joining of contigs at one end of the scaffold that includes fewer short-range interactions at the diagonal and an absence of long-range interactions elsewhere in the chromosome off the diagonal (upper left of diagonal). These contigs were removed for further analysis. Contig boundaries in the assembly are denoted by the black triangles along the diagonal.

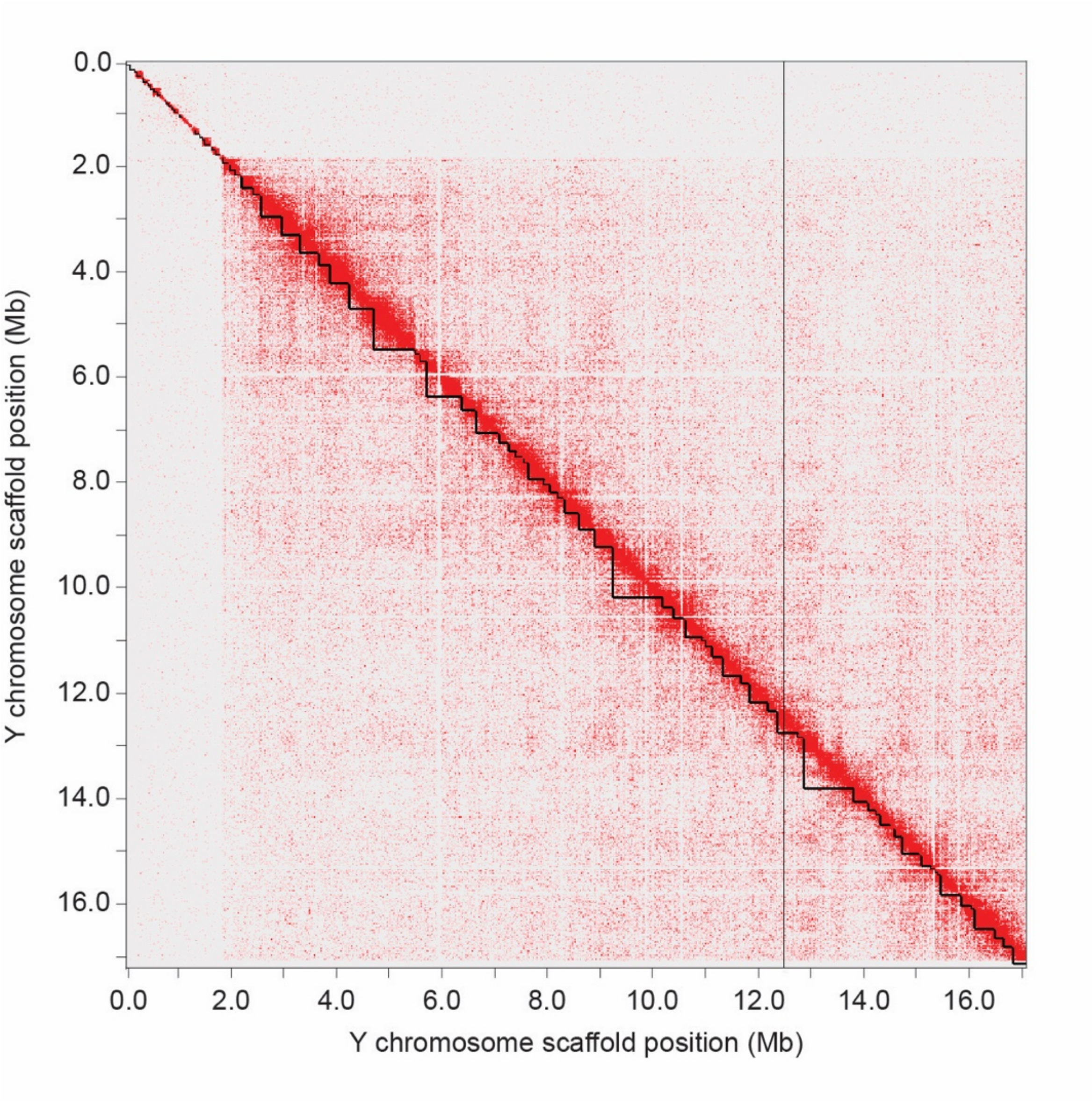

Figure S4. Stratum one is located on the end of the Y chromosome, opposite of *Idh*. Fluorescent *in situ* hybridization probes were generated from a stratum one BAC (CHORI 215-013C20). The BAC (pink) is located at the end of the Y chromosome (arrow), opposite from *Idh* (green) on mitotic metaphase spreads. *Idh* is located centrally on the X chromosome (arrowhead).

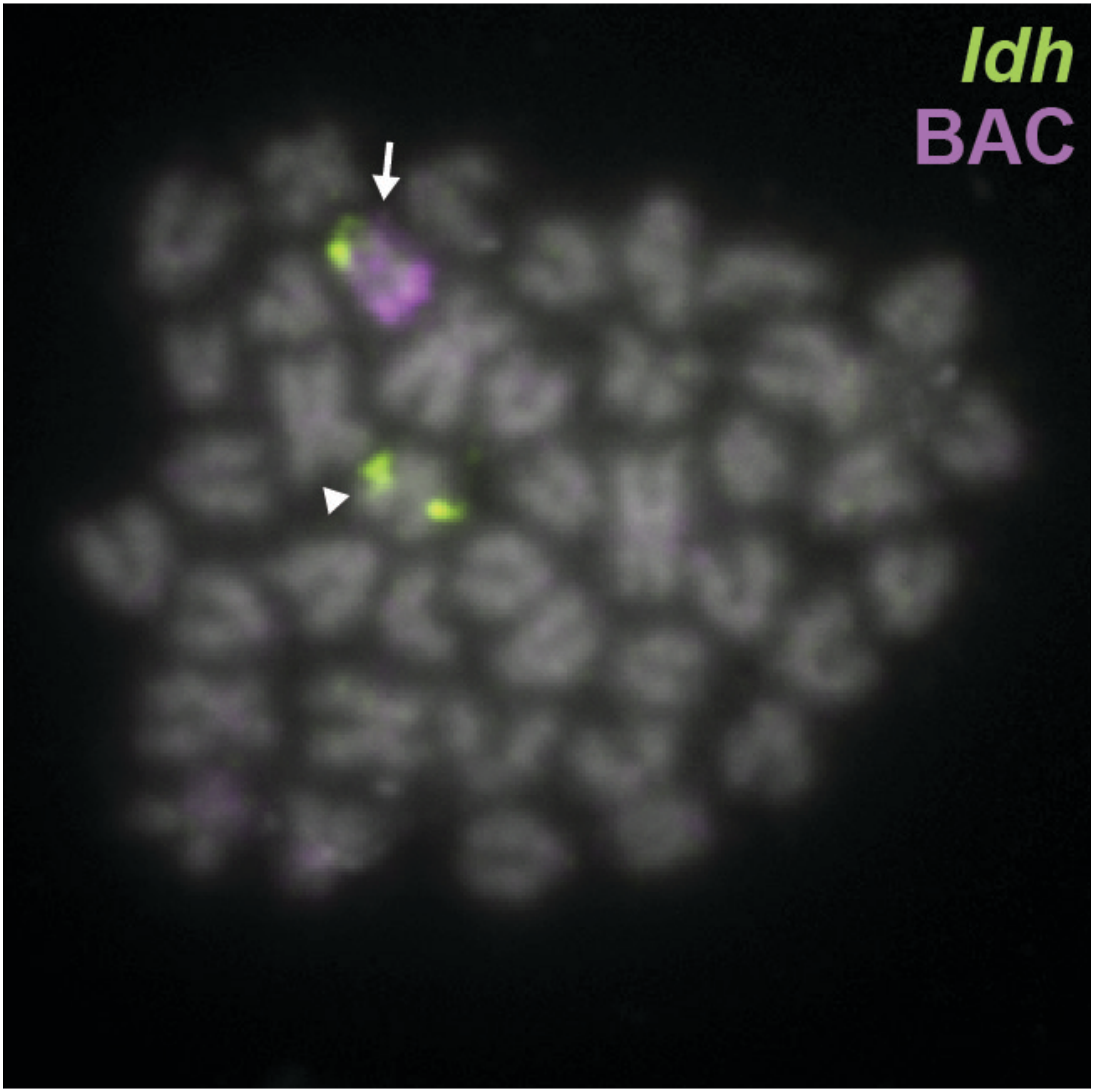

Figure S5. Short-read sequences aligned to the X Chromosome show half coverage in males and full coverage in females. Read depth is shown normalized to autosome coverage. Each point is a 1 kb window across the chromosome. The red dashed line shows median coverage across the non-recombining region (strata one, two, and three combined).

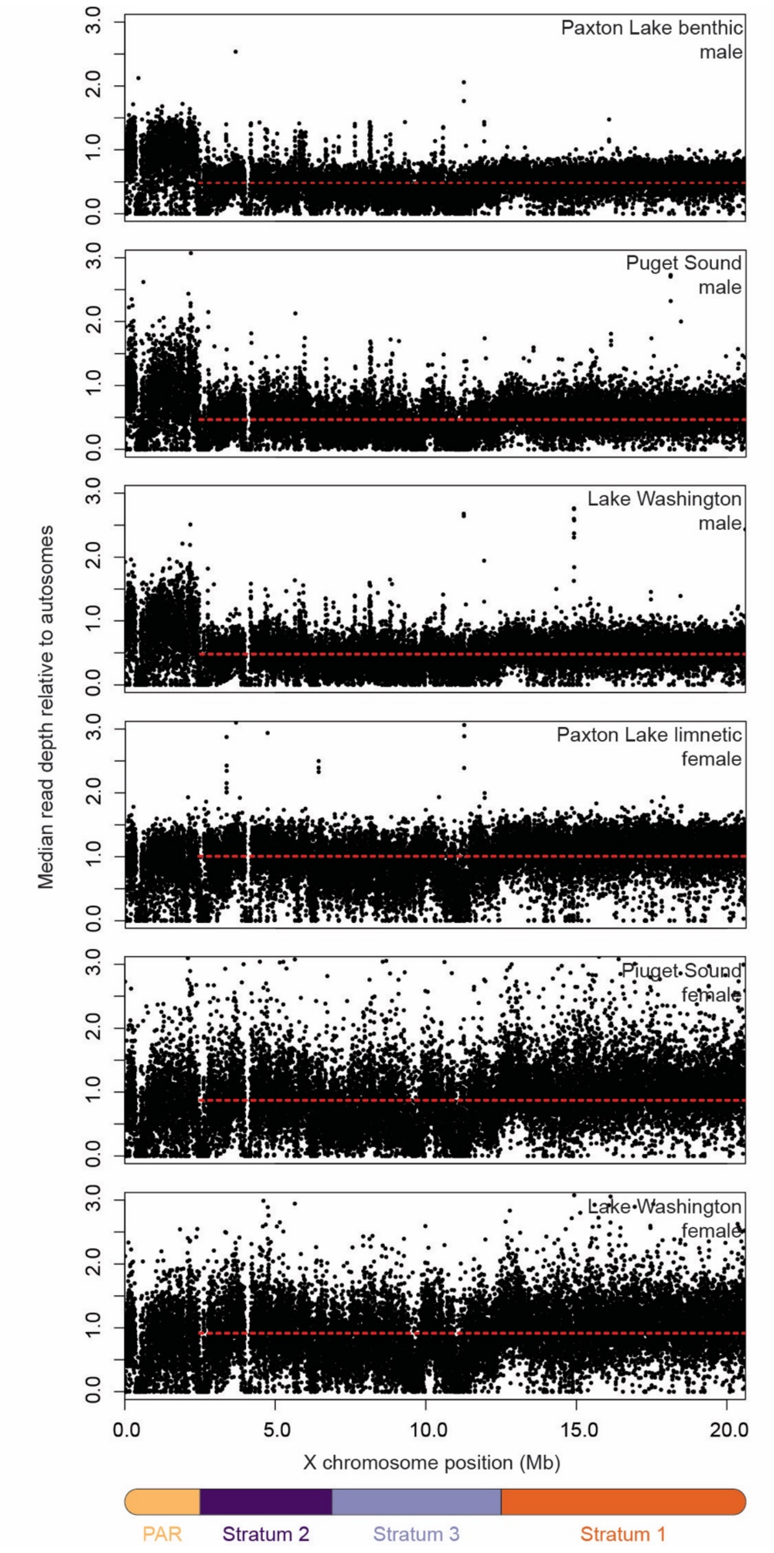

Figure S6. Short-read sequences aligned to the Y Chromosome show half coverage in males and no coverage in females. Read depth is shown normalized to autosome coverage. Each point is a 1 kb window across the chromosome. The red dashed line shows median coverage across the non-recombining region (strata one, two, and three combined).

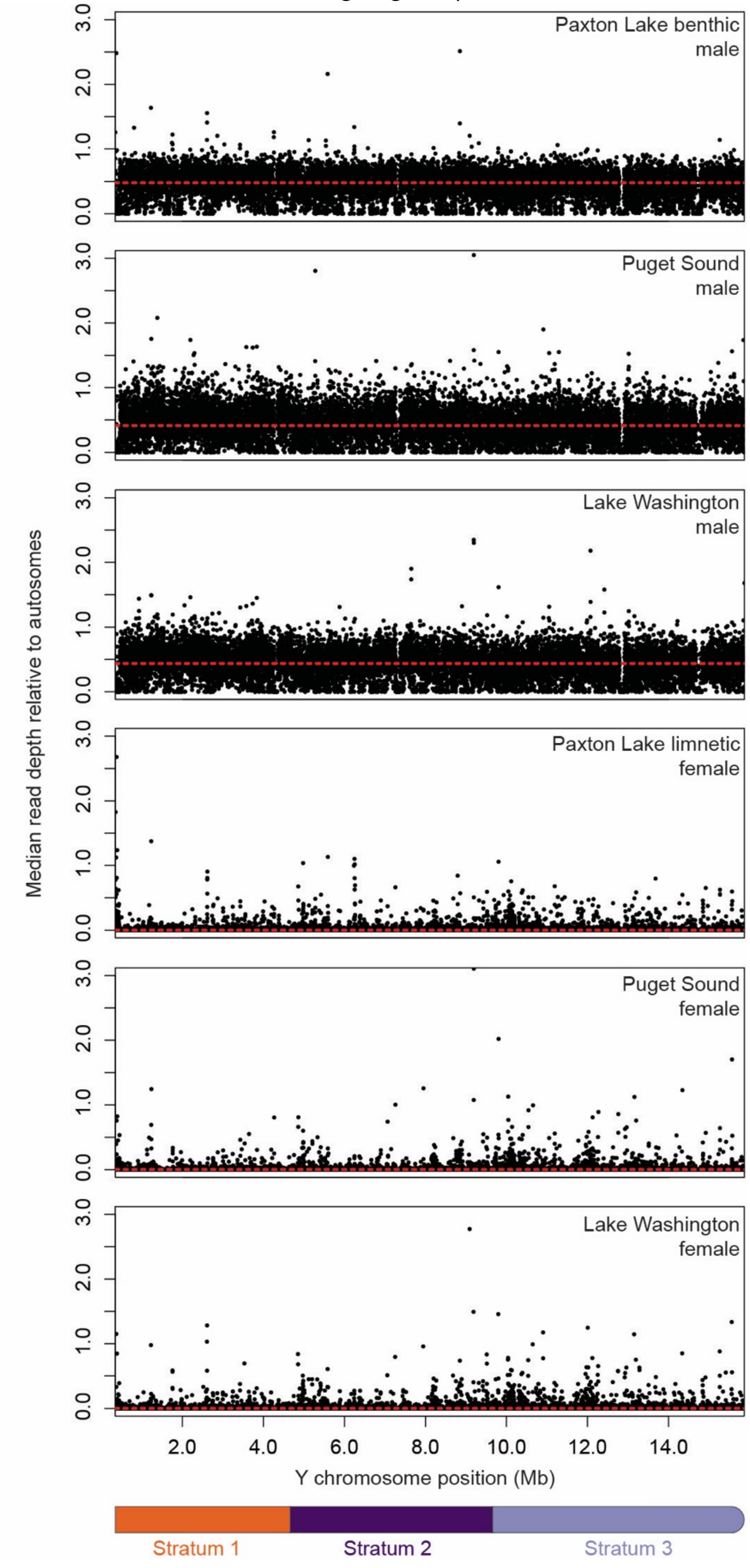

Figure S7. Median coverage of short-read sequences in females and males of three different populations. Read depth was measured in 1 kb windows across the chromosomes and normalized to autosome coverage. Males show half coverage on the X and Y chromosomes, whereas females have full coverage on the X chromosome and no coverage on the Y chromosome.

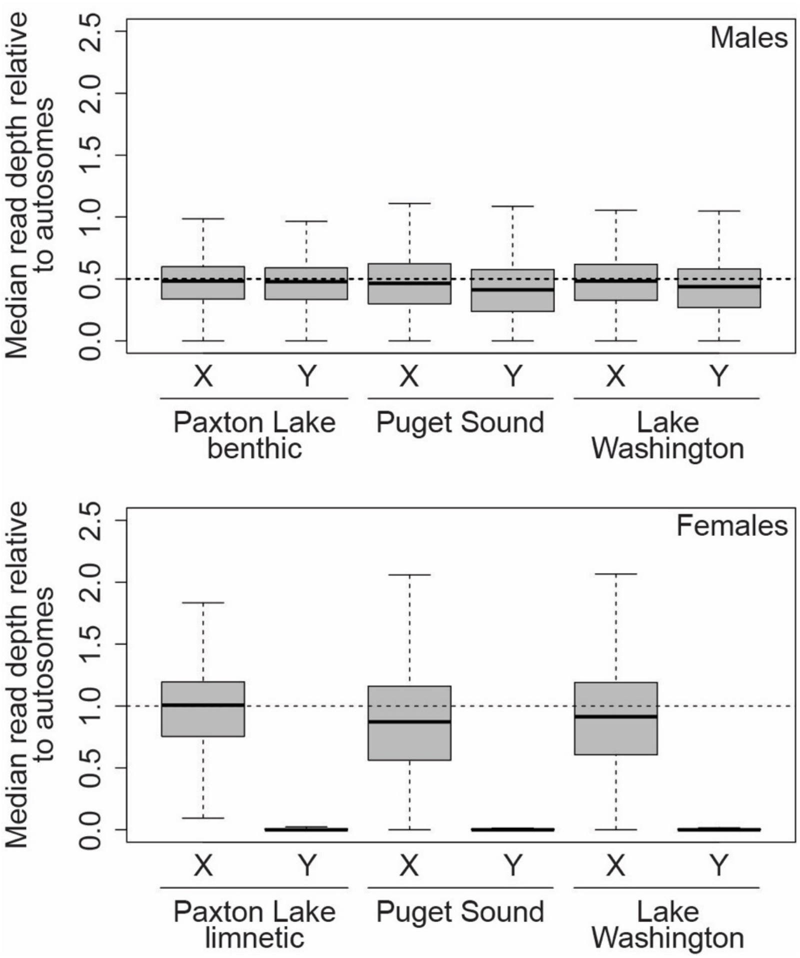

Figure S8. Short-read sequences from a chromatin-immunoprecipitation (ChIP-seq) with CENP-A from a second male fish were aligned to the reference Y chromosome assembly. There is a prominent peak between markers STN187 and WT1A where the centromere is located.

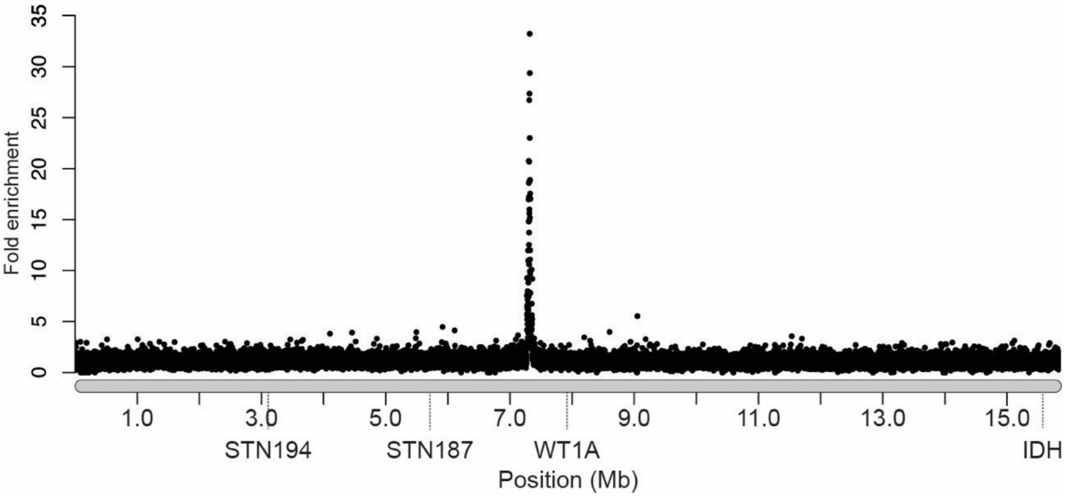

Figure S9. Alpha satellite monomeric repeats found on the Y chromosome show conservation with the core centromeric repeat found on the autosomes and the X chromosome. The putative CENP-B Box is shown in red.

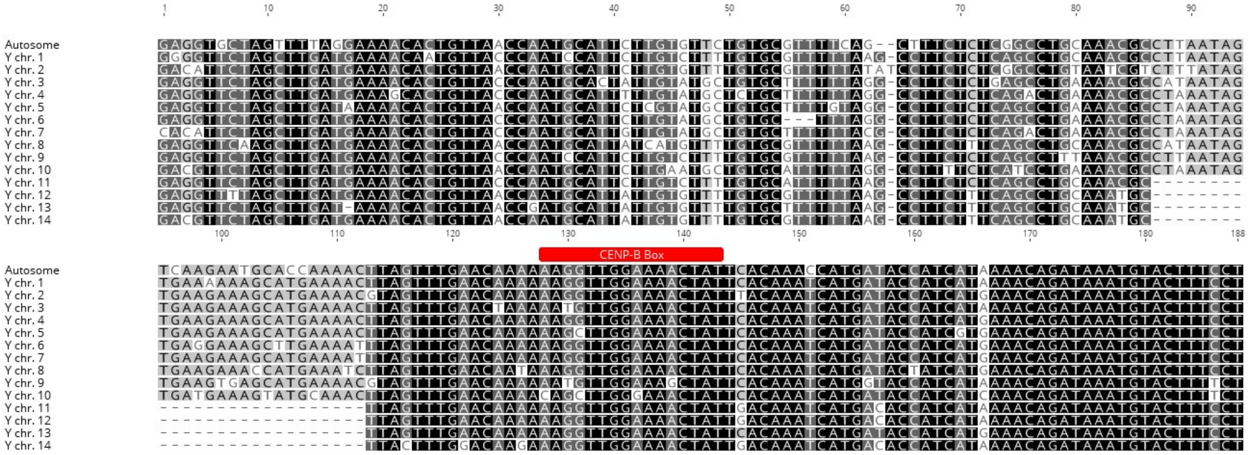

Figure S10. Transposable elements are at a higher density across the Y chromosome relative to the X chromosome. Repeat families were identified using a combination of RepeatModeler and RepeatMasker. The total number of repeats across 250 kb bins are shown.

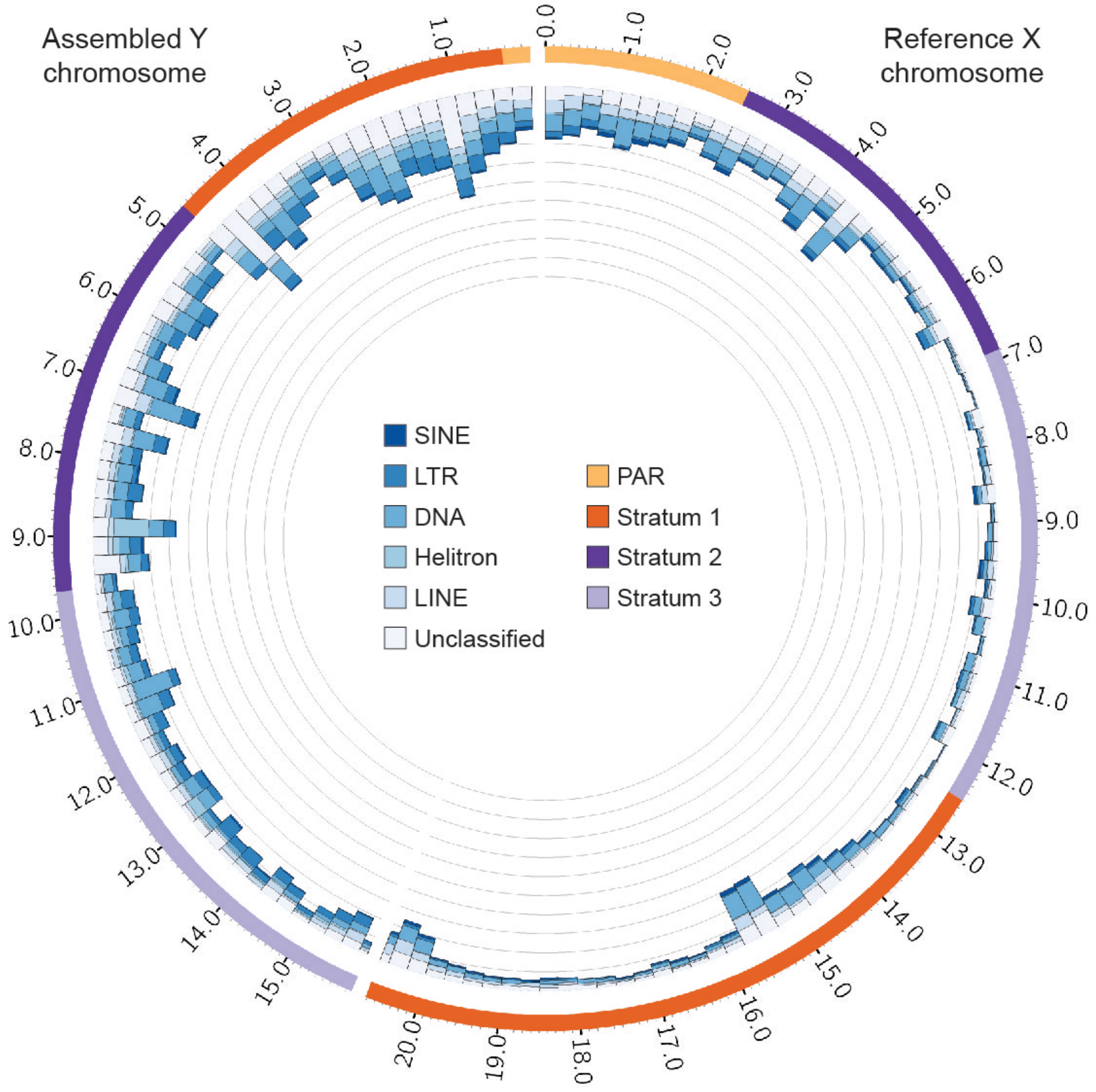

Figure S11. Transposable elements are at higher densities in each stratum of the Y chromosome compared to the X chromosome. Each transposable element family shown in Figure S10 is included in the summary.

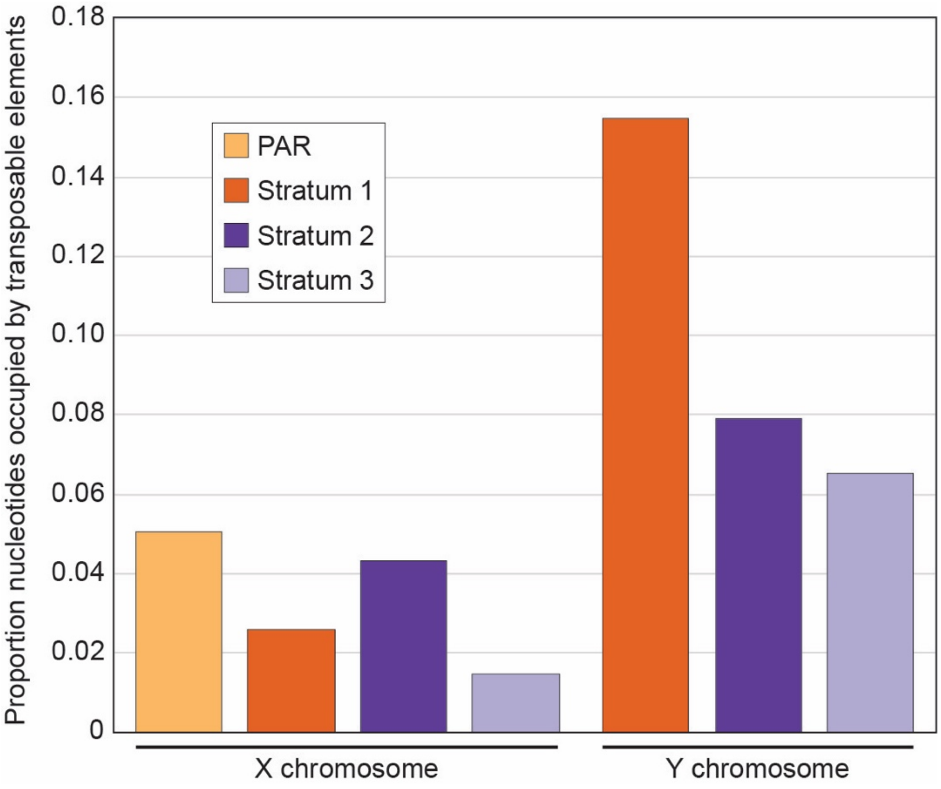

Figure S12. Alignment of protein coding sequences of vertebrate *Amh* genes. Conserved residues across taxa are highlighted in black. The Amh and TGF-β functional domains are shown in red.

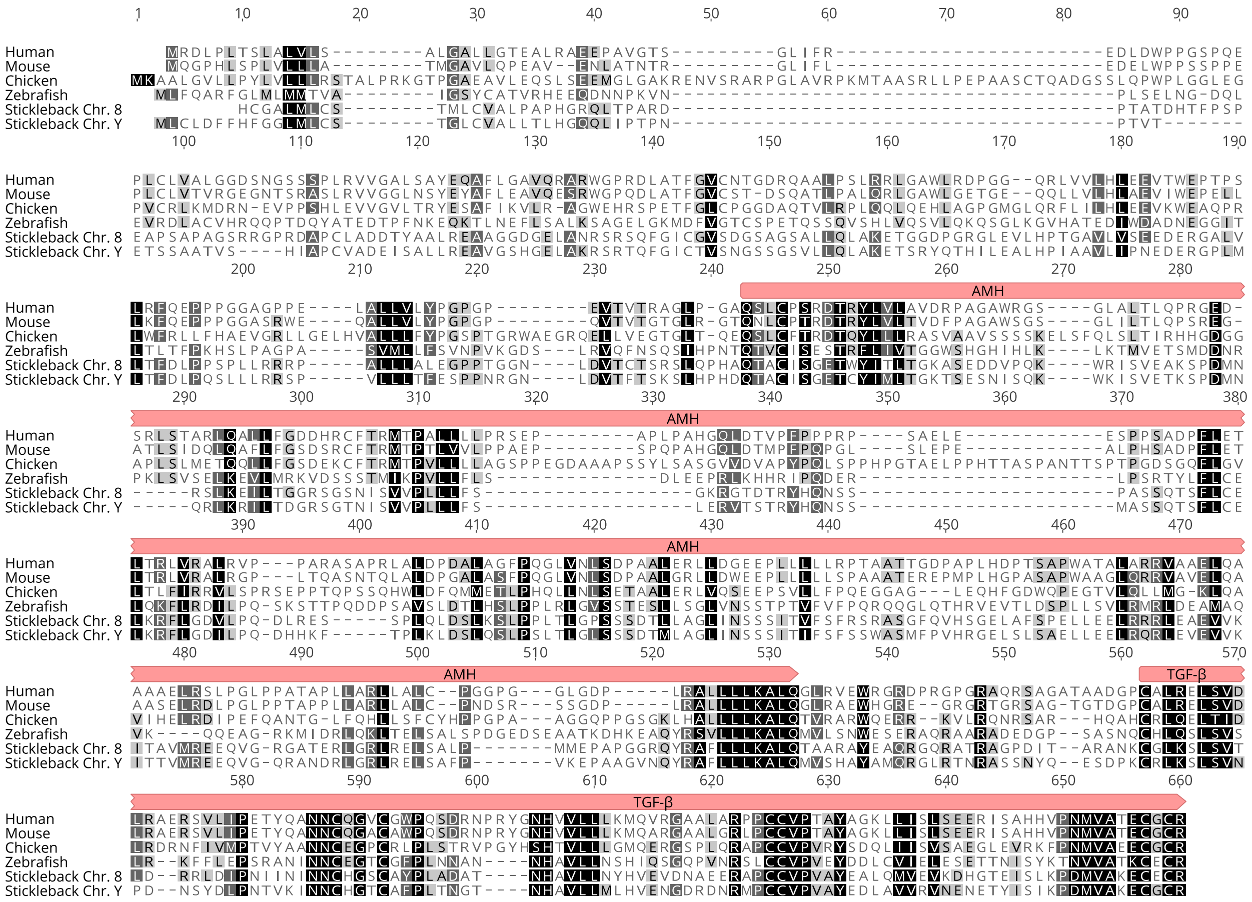

Supplement: Supplementary file 2 — Additional file 2. Supplementary figures. [file 13059_2020_2097_MOESM2_ESM.pdf]
